# Inhibition of Neutrophil Collagenase/MMP-8 and Gelatinase B/MMP-9 and Protection against Endotoxin Shock

**DOI:** 10.1155/2014/747426

**Published:** 2014-11-26

**Authors:** Zheng Qiu, Jianghai Chen, Hanmei Xu, Philippe E. Van den Steen, Ghislain Opdenakker, Min Wang, Jialiang Hu

**Affiliations:** ^1^School of Life Science and Technology, China Pharmaceutical University, Nanjing 210009, China; ^2^Department of Hand Surgery, Union Hospital of Tongji Medical College, Wuhan 430022, China; ^3^The Key Laboratory of Modern Chinese Medicines, Ministry of Education, China Pharmaceutical University, Nanjing 210009, China; ^4^Laboratory of Immunobiology, Rega Institute for Medical Research, University of Leuven, Minderbroedersstraat 10, 3000 Leuven, Belgium

## Abstract

Endotoxin shock is a life-threatening disorder, associated with the rapid release of neutrophil enzymes, including neutrophil collagenase/matrix metalloproteinase-8 (MMP-8) and gelatinase B/matrix metalloproteinase-9 (MMP-9). After activation, these enzymes cleave extracellular matrix components and cytokines and thus may contribute to shock syndrome development. MMP inhibitors have been suggested as immunotherapy of endotoxin shock. However, little is known about the therapeutic time window of MMP inhibition. Here, a sublethal endotoxin shock mouse model was used to evaluate the effect of an MMP inhibiting peptide (P2) after intravenous or intraperitoneal injection and to study the time window between LPS and inhibitor injections. With the use of a specific ELISA the plasma P2 concentrations were monitored. Whereas we corroborated the treatment strategy of MMP targeting in endotoxin shock with a new inhibitor, we also demonstrated that the time window, within which effective MMP inhibition increased the survival rates, is rather limited.

## 1. Introduction

Bacteremia and septic shock are among the most frequent causes of mortality in modern hospitals [1]. These diseases are most often caused by bacterial superinfections or their products, for instance, endotoxin, and form clinical examples of severe immunopathology [2, 3]. Endotoxin shock induction is a commonly used animal model to evaluate the protective effect of biologically active agents. During shock syndrome development, lipopolysaccharide (LPS) activates the inflammatory response by binding to Toll-like receptors (TLR) on multiple leukocyte types [4]. Excessive TLR activation leads to exaggerated stimulation of leukocytes and excessive production of inflammatory mediators, including cytokines and enzymes [5, 6]. Since neutrophils are the most abundant white blood cell type in the human circulation, LPS will mainly, directly, and immediately act on these cells. This interaction results in the release of neutrophil effector molecules, including enzymes and reactive oxygen intermediates that contribute to the activation of MMPs [7]. An important aspect of septic shock is its acuteness which may be based on the fast release of mediators by degranulation [5, 6]. In human volunteers and primate models, MMP-9 plasma levels were already maximal at 1.5 to 3 hours after LPS challenge [3, 5]. Effective inhibition of MMP activities during the early stage of endotoxin shock syndrome development by the peptides Regasepin 1 and Regasepin 2 has been documented [8, 9].

The peptide P2 was previously defined as an antitumor peptide and is formed by the connection of an MMP-inhibiting peptide sequence (Inhibitor 2) to the N-terminus of an endostatin fragment, named ES-2 [10]. ES-2 represents 60–70 amino acids of endostatin and has antiangiogenic activity. Inhibitor 2 was designed based on the backbone of the previously described MMP-inhibitory peptides Regasepin 1 and Regasepin 2, by targeting MMP-9 and TNF-alpha converting enzyme (TACE) [11]. The fusion peptide P2 has a similar inhibitory profile against MMP activities as Inhibitor 2 [10]. HM-3 is another antitumor peptide [12]. It was formed by the connection of the RGD sequence to the C-terminus of ES-2. HM-3 has a short* in vivo* half-life of only 27 minutes [12] (peptide sequences of the above-mentioned peptides in Table 1).

Several methods exist for the determination of protein and peptide concentrations in plasma, for example, high-performance liquid chromatography (HPLC) [13], gas chromatography (GC) [14], capillary electrophoresis (CE) [15], radioimmunoassay (RIA) [16], and mass spectrometry [8]. However, these methods are labour-intensive for sample pretreatment and require the availability of expensive detectors. Enzyme-linked immunosorbent assay (ELISA) is an affordable and simple immunological detection method in the field of preclinical and clinical analysis of protein and peptide drugs [17, 18]. Therefore, to monitor the pharmacokinetics of P2 in order to define the therapeutic window for the treatment of endotoxin shock by MMP inhibition, we also developed a competition ELISA for this peptide inhibitor.

## 2. Materials and Methods

### 2.1. Reagents and Animals

Peptide P2 and Inhibitor 2 (more than 96% purity) were chemically synthesized by GL Biochem Ltd. (Shanghai). Bovine serum albumin (BSA) and human serum albumin (HSA) were purchased from Sigma. P2-BSA was used as the ELISA coating antigen. It was produced by linking of P2 to BSA by reaction with a carbodiimide reagent (kit 786-068, Boyuan Biotechnology, China). Horseradish peroxidase-labelled goat anti-rabbit secondary antibody was purchased from Multi-Sciences Co., Ltd., China.

Adult female Swiss mice (6-7 weeks, 18–20 g) were purchased from the Shanghai Animal Center of the Chinese Academy of Sciences and used under the experimental animal production license: SCXK (Hu) 2012* *0004. All animals were housed in a controlled environment (25°C; 12 h light-dark cycle), with water and food provided freely. The authors confirm that experiments involving animals adhered to the institutional ethical standards of China Pharmaceutical University and the care of animals was independently assessed and approved in accordance with the licensing guidelines of China Pharmaceutical University.

### 2.2. Establishment of Endotoxin Shock Model

#### 2.2.1. LD50 Determination and Intravenous Administration of Inhibitors

Prior to formal experiments, the dosis yielding 50% lethality (LD50) was determined. Adult female Swiss mice were placed in five groups with 4 mice per group. Mice in Groups 1 to 5 were intravenously injected with 100 *μ*L (0.5, 1, 2, 3, or 4 *μ*g/*μ*L) LPS from* E. coli* serotype 0111:B4 (Sigma) dissolved in 0.9% saline, respectively. At 24 hours after administration, the survival rates in each group were observed and the LD50 of the used Swiss mice treated with LPS was obtained. Similar results were obtained in independent experiments and various production lots of LPS (Lot number 091M4031V, Lot number 012M4098V, and Lot number 099K4025) yielded, respectively, LD50 values of 100, 50, and 50 *μ*g per mouse.

As Regasepin 2 (peptide sequence in Table 1) was found to protect mice from endotoxin shock by intravenous injection of 100 *μ*L (7 mg/mL) Regasepin 2 (35 mg/kg) 5 minutes after intravenous injection of LPS [9], it was included here as a positive control peptide. The protective effect of all inhibitors was evaluated in a similar way. P2 (90 mg/kg P2) was used at a similar molar concentration as Regasepin 2. The effect of lower dosages of P2 (30 mg/kg and 10 mg/kg) was also evaluated in parallel experiments. The details of the animal experiments, including grouping of mice, number of mice in each group, reagent concentrations, and ways of administration, are shown in Table 2. The peptides were administered intravenously 5 minutes after LPS injection.

The protective effects via intravenous injection were evaluated in groups of 6 mice, intravenously injected with 100 *μ*L (7 mg/mL; 35 mg/kg) Regasepin 2 or Inhibitor 2 (35 mg/kg) 5 minutes after intravenous injection of LPS (200 *μ*g per mouse). The numbers of surviving mice were recorded regularly after LPS administration. Kaplan-Meier survival curves were generated. *P* values were calculated compared with the survival rates of the negative control group. The experiments were performed two times.

#### 2.2.2. Evaluation of the Protective Effect of P2 via Intraperitoneal Administration

The experimental details are shown in Tables 3 and 4. Regasepin 2 via intravenous injection (35 mg/kg) was used as a positive control peptide. 200 *μ*L P2 (15 mg/mL) was administered intraperitoneally (150 mg/kg) 30, 60, or 90 minutes before intravenous LPS challenge (200 *μ*g per mouse) to evaluate the effect of P2 via this administration route. The protective effects of a single intravenous injection of P2 (10 mg/kg) and two times intravenous administration of P2 (10 mg/kg) with 2 hours interval were also included and compared.

### 2.3. Establishment of Indirect Competitive ELISA

#### 2.3.1. Generation of Polyclonal Antibodies Recognizing P2

New Zealand white rabbits were subcutaneously immunized with a conjugate between P2 and keyhole limpet hemocyanin (P2-KLH) to produce the polyclonal antibodies. Freund complete adjuvant (FCA) was employed in the first immunization and Freund incomplete adjuvant (FIA) was used in the subsequent booster injections. Rabbits were immunized every three weeks with 500 *μ*g of immunogen, and blood samples from the marginal vein of the ear were taken for identification of seroconversion. Ten days after the final boost, both rabbits were exsanguinated by heart puncture and the serum was separated from blood cells by storing at 4°C overnight and centrifugation at 500 rpm for 10 min. Then the antibodies in this crude serum were purified using Protein-G affinity column and the purified antibody was used to setup the indirect ELISA method.

#### 2.3.2. Development of a Competition ELISA for P2

The concentration of P2-BSA that was used to coat the plates and the reagent concentration of the P2-specific polyclonal antibody were optimized by two-dimensional serial dilution tests. 96-well microtiter plates were coated with a solution of 6 *μ*g/mL P2-BSA in 0.05 M sodium bicarbonate (pH 9.6) and the plate was kept overnight at 4°C. After washing the plate three times, the plate was blocked with 0.1 M PBS (pH 7.4) containing 10% (V/V) skim milk at 37°C for 1.5 h. To perform the competition ELISA between P2 and the immobilized P2-BSA, P2-specific antibody (1000 times serum dilution) was added to the wells in the presence of plasma or standard samples at 37°C for 1.5 h. After washing the plate three times, 100 *μ*L of horseradish peroxidase-conjugated goat anti-rabbit IgG (1 : 2000) was added and incubated for 1 h at 37°C. After washing three times, the tetramethylbenzidine (TMB) substrate (100 *μ*L/well) was added and the reaction was executed for 15 min. The reactions were stopped with 2 M sulfuric acid (50 *μ*L/well). The absorbance of each well at 450 nm was detected with an automated ELISA reader. The calibration curve was expressed according to the fact that 1-B/B0 are linear with log C (where B0 is the OD value in the absence of P2 in plasma, B is the OD value at serial concentrations of P2 in plasma, and C is the P2 concentration, resp.). The indirect ELISA procedures were conducted in duplicate with a set of standard concentrations of P2 in diluted mouse serum in order to determine the mathematical fitting equation and linear detection range. Human serum albumin (HSA) and bovine serum albumin (BSA) were used to detect the specificity of the P2 antibody. Serial dilutions (1 ng/mL, 10 ng/mL, 100 ng/mL, 1 *μ*g/mL, 10 *μ*g/mL, 100 *μ*g/mL, and 1 mg/mL) of the selected proteins were measured by indirect ELISA. Calibrations of 1-B/B0 versus log C were done, with, in these respective conditions, C being the concentration of BSA or HSA.

### 2.4. Pharmacokinetics Study of P2

#### 2.4.1. Sample Collection

Adult female Swiss mice, 5 animals per group, were used for the pharmacokinetic studies. P2 (90 mg/kg, 30 mg/kg, or 10 mg/kg) was dissolved in sterile 0.9% saline and was administered intravenously. Blood samples were collected through the canthus at 0, 3, 6, 9, 12, 15, and 30 min. For intraperitoneal injection of P2, 150 mg/kg P2 dissolved in 0.9% saline was administered and blood samples were collected through the canthus at 0, 13, 20, 40, 68, 100, 127, and 168 minutes.

#### 2.4.2. Sample Pretreatment

Blood samples were immediately centrifuged (12000 rpm for 30 seconds), and the supernatants were taken and diluted 10, 30, or 300 times with PBS, prewarmed at 80°C in a water bath. The diluted samples were kept at 80°C for another 30 min to remove enzyme activity of the plasma components. Samples were diluted with prewarmed PBS so that the final concentration of P2 was within the linear range of the standard line. After another centrifugation (12000 rpm for 2 min), the diluted samples were analyzed by ELISA to measure the plasma P2 concentrations.

### 2.5. Statistical Methods

Results were represented as mean ± SD. Statistical significance was assessed using statistics software SPSS13.0. Two-way analysis of variance (ANOVA) was used to test for differences between groups. For all statistical comparisons, positive groups were compared with negative controls, and ^*^ *P* < 0.05 or ^**^ *P* < 0.01 were considered statistically significant.

## 3. Results

### 3.1. Protection from Endotoxin Shock after Intravenous P2 Injection

**Figure 1:**
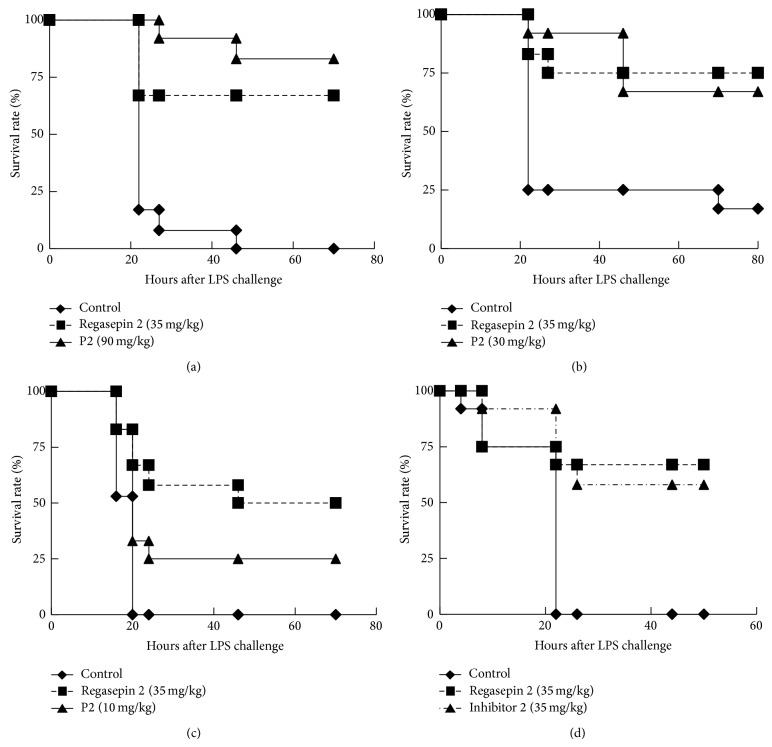
P2 and Inhibitor 2 protect Swiss mice from endotoxin shock after intravenous administration. In all the experiments, 35 mg/kg Regasepin 2 by intravenous injection was included as a positive control peptide. The protective effects of P2 at a dosage of 90 mg/kg (a), 30 mg/kg (b), and 10 mg/kg (c) by intravenous injection were shown. (d) The effect of 35 mg/kg Inhibitor 2 by intravenous injection was evaluated in a similar way as in panel (a) to (c). Kaplan-Meier survival curves were generated. The results of two parallel and independent experiments were pooled (*n* = 12 for each condition). The details of the animal experiments were tabulated in Table 2. All peptides and 0.9% NaCl control solution were administrated intravenously 5 minutes after LPS injection.

In endotoxin shock models, the survival rate might be influenced by LPS preparations. LD50 values of a few LPS preparations for the same mice strain were determined by incremental dosing. One specific batch (Lot number 091M4031V) with an LD50 of 100 *μ*g per mouse was used for the subsequent animal work. Groups of 6 mice were intravenously injected with P2 or Regasepin 2 5 minutes after intravenous injection of LPS (200 *μ*g per mouse). The mice were observed at regular intervals. As expected, in three independent experiments, Regasepin 2 at 35 mg/kg displayed a significant protective effect in Swiss mice after intravenous administration (35 mg/kg) and the *P* value, compared with the corresponding negative control group, was below 0.0005 (Figure 1(a)), 0.013 (Figure 1(b)), and below 0.0008 (Figure 1(c)). P2 at a dose of 90 mg/mL also showed a significantly protective effect with a *P* value of 9.13 × 10^−7^ compared with the negative control group (Figure 1(a)). Furthermore, P2 at a dose of 30 mg/kg also showed a significant protective effect (*P* = 0.0054; Figure 1(b)). At a dose of 10 mg/kg (although less mice survived in G3 than in G2 (see the tables)), P2 still showed a significant protective effect (*P* = 0.0138) (Figure 1(c)).

As Inhibitor 2 has a similar structure and inhibitory profile as Regasepin 2, the protective effect of Inhibitor 2 in the endotoxin shock model was also evaluated. As shown in Figure 1(d), all mice in the negative control group died 22 hours after LPS injection and Regasepin 2 and Inhibitor 2 both showed a significant protective effect (*P* = 0.0087 and 0.0016, resp.).

### 3.2. Establishment of Indirect Competitive ELISA

**Figure 2:**
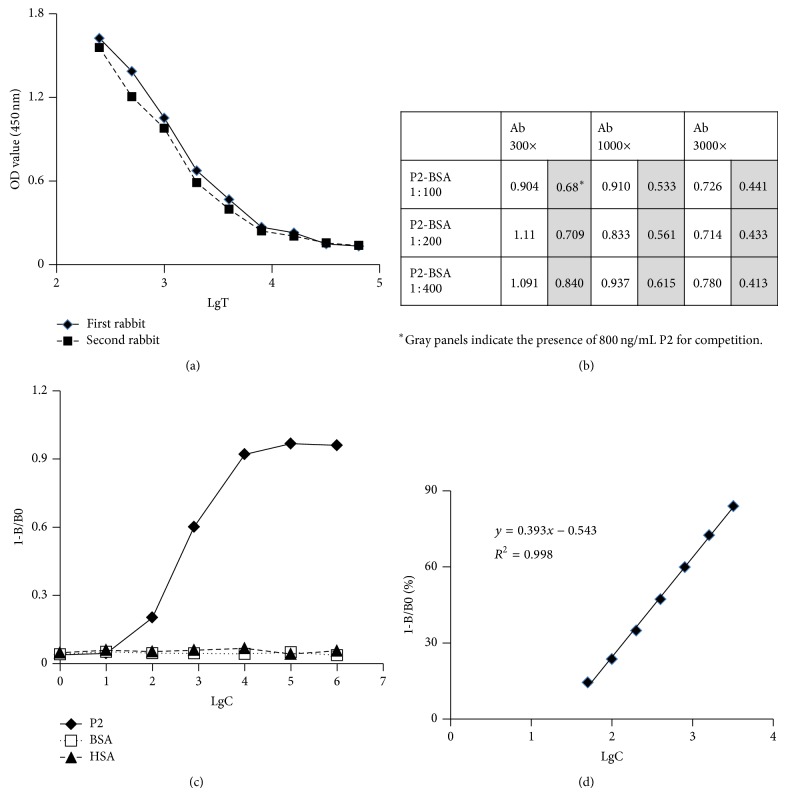
Establishment of the indirect ELISA for detection of plasma P2 concentrations. (a) Immunoreaction of purified antibody from two rabbits that were immunized with P2-KLH. LgT represents the logarithm of serum dilution factors. (b) Two-dimensional serial dilution method for selection of coating concentration of P2-BSA and reagent concentration of polyclonal anti-P2 antibody. Two columns of data exist under each antibody dilution condition. The white panels represent absorbance after binding of the antibody to the immobilized P2-BSA whereas the gray panels represent absorbance after binding of the antibody to immobilized P2-BSA in presence of 800 ng/mL P2 as a competitor. (c) Antibody specificity determination. (d) The standard curve with P2 concentrations ranging from 50 to 3200 ng/mL. B0 is OD value in the absence of P2 in plasma, B is OD value at serial concentrations of P2 in plasma, and C is P2 concentration. In this example, serial P2 concentrations were prepared in mouse plasma that had been diluted 30 times with prewarmed PBS at 80°C.

In order to validate MMP inhibition* in vivo*, it is critical to monitor that the inhibitor is present at sufficient concentrations. Two rabbits were immunized with P2-KLH. The purified antibody was used to establish a competition ELISA to detect P2 levels. From Figure 2(a), it was deduced that the purified antibodies from the two rabbits showed similar binding graphs to the immobilized P2-BSA. The antibody from the first rabbit was used in the following experiments. Two-dimensional serial dilution was performed to select the proper coating concentration of P2-BSA and the reagent concentration of anti-P2 polyclonal antibody. From Figure 2(b), the combination of P2-BSA (1 : 100 dilution) and the antibody (1 : 1000 dilution) gave not only a good absorption but also a good competition by 800 ng/mL P2. The dilution for the HRP-labeled goat anti-rabbit secondary antibody was determined to be 1 : 2000. Furthermore, from Figure 2(c), P2 showed a good competitive inhibition of antibody binding to the immobilized P2-BSA, whereas HSA and BSA showed no inhibition even at high concentrations. These data confirmed that the generated antibody against P2 does not cross-react with other selected proteins. Calibration curves for determination of P2 by the indirect competitive ELISA were established. Satisfactory curves were always obtainable between 50 and 3200 ng/mL of P2 concentrations. A typical standard curve is shown in Figure 2(d). The parameter 1-B/B0 correlated well with log C (*Y* = 0.393*X* − 0.543, *R* ^2^ = 0.998). In this example, P2 was diluted with decomplemented mouse serum that was diluted 30 times with PBS.

### 3.3. Pharmacokinetic Analysis of P2 in Swiss Mice after Intravenous Injection

**Figure 3:**
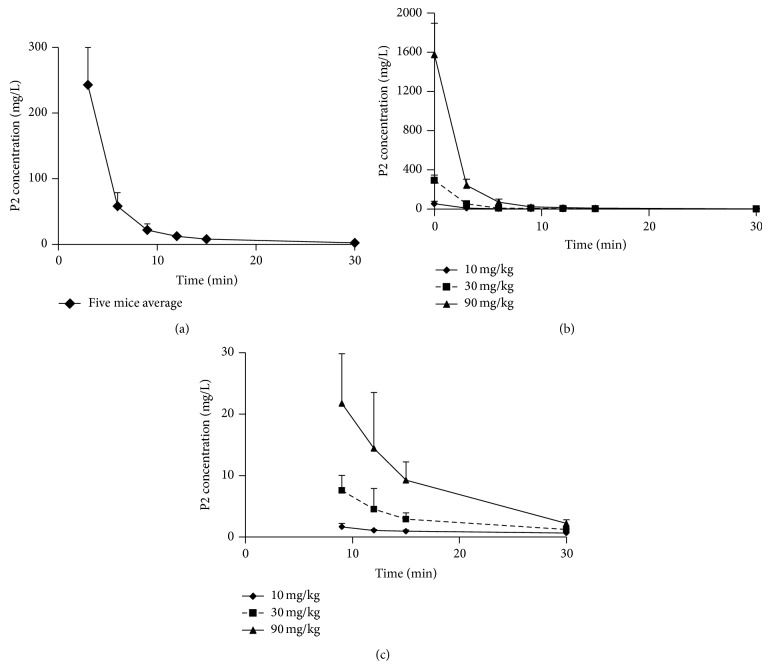
Plasma concentrations of P2 versus time after an intravenous bolus injection. (a) The profiles of P2 at a dose of 90 mg/kg in five individual mice were averaged. The data point at time 0 is not shown. (b) The profiles of P2 plasma concentrations versus time after intravenous injection of 90, 30, or 10 mg/kg P2 were shown. (c) For a clear observation of the profiles in (b), the data from time point 9 to 30 minutes were magnified.

We applied the competitive ELISA for a pharmacokinetic analysis of samples that were collected after intravenous administration of P2. After a single intravenous injection of 100 *μ*L (17.7, 5.9, or 2.0 mg/mL) P2, which corresponded to the dosage of 90, 30, and 10 mg/kg (see Figure 1), we monitored the drug concentrations in plasma until 30 min after administration. In Figure 3(a), the profiles of P2 at a dose of 90 mg/kg, averaged from five individual mice, are shown. In Figure 3(b), the profiles of P2 concentration* versus* time after intravenous injection of P2 are shown. Intravenous administration of P2 produced an immediate peak in plasma P2 levels in mice. The plasma P2 level dropped rapidly within 6 min after injection and then declined slowly and progressively. For a better observation, the profiles for the three dosages from 9 to 30 minutes were shown in Figure 3(c). The higher dosing resulted, as expected, in higher inhibitor concentrations. This result is in accordance with the survival results in Figures 1(a)–1(c). From Figure 3(c), it can be deduced that, after injection of 100 *μ*L (2 mg/mL) P2, with minimal protective effect (*P* = 0.0138, Figure 1(c)), an effective P2 concentration (above 2 *μ*M) still remained in plasma for 6 minutes. This indicated that, for the used animal model of endotoxin shock, an effective inhibition of the relevant enzymes within the first 11 minutes (a six-minute degradation time and a five-minute interval between LPS and P2 administration) after LPS administration can successfully alleviate shock syndromes and increase survival rate of experimental mice. These data illustrate that protease inhibition during an extremely early and short time interval is sufficient for therapeutic efficiency in endotoxin shock.

### 3.4. Endotoxin Shock after Intraperitoneal Injection of P2

**Figure 4:**
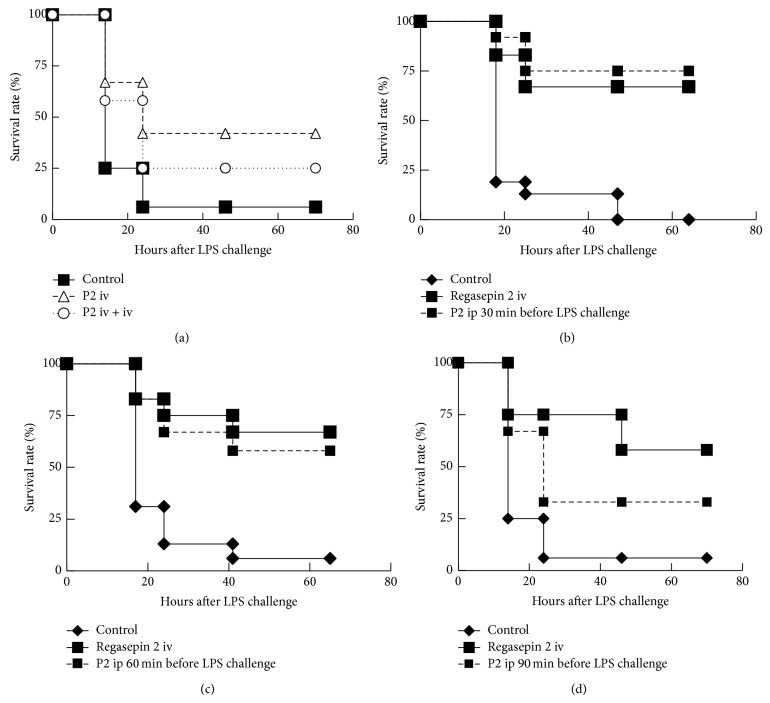
P2 protects Swiss mice from endotoxin shock after intraperitoneal administration. For all the experiments, 35 mg/kg Regasepin 2 via intravenous injection was included as a positive control. (a) A single intravenous injection of 10 mg/kg P2 protected mice from endotoxin shock, whereas two injections of 10 mg/kg P2 at two hours interval decreased the survival rate of the mice. The protective effects of P2 at a dosage of 150 mg/kg via intraperitoneal injection 30 minutes (b), 60 minutes (c), and 90 minutes (d) before intravenous injection of LPS (200 *μ*g per mouse) were shown. Kaplan-Meier survival curves were generated. The results of two parallel and independent experiments were pooled. The details of the animal experiments were tabulated in Tables 3 and 4.

The experimental strategies are shown in Tables 3 and 4. In Figure 4(a), P2-treated mice showed a significant difference with the LPS-treated control group, indicating that P2 at 10 mg/kg protected mice from endotoxin shock. A low dose of 10 mg/kg was selected here to study longer inhibition intervals in the case that the second intravenous injection of P2 provided further protective effect. The rationale of the experimental design (in G3) with two intravenous injections was indeed to prolong P2 concentrations at inhibitory levels in the circulation. However, the result showed that less mice survived (in G3 than in G2). This experiment was repeated two times with the same result. This implied that a second intravenous injection of P2 did not improve the outcome. In Figures 4(b), 4(c), and 4(d), groups of 6 mice were injected intraperitoneally with P2 (150 mg/kg) 30, 60, or 90 minutes before LPS challenge. As expected, Regasepin 2 via intravenous administration showed a significant protective effect. In the corresponding experiments, P2 via intraperitoneal injection showed significant protective effects (*P* < 0.0004, 30 minutes before LPS challenge; *P* = 0.0011, 60 minutes before LPS challenge; *P* = 0.0243, 90 minutes before LPS challenge). The mouse survival data indicated that P2 via intraperitoneal injection showed better protective effect when injection intervals between P2 and LPS decreased. All the experiments were performed two times with reproducible results.

### 3.5. Pharmacokinetic Analysis of P2 in Swiss Mice after Intraperitoneal Injection

**Figure 5:**
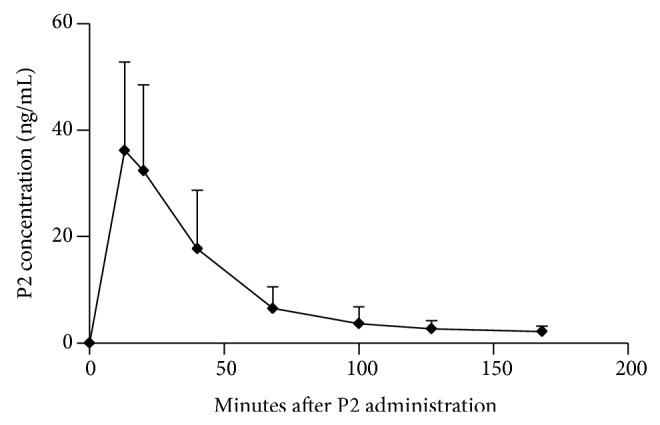
Plasma concentrations of P2 versus time after a single intraperitoneal injection. The profiles of P2 (150 mg/kg) in five individual mouse were averaged.

The developed indirect ELISA was also used for a pharmacokinetic analysis of samples that were collected after intraperitoneal injection of P2 (150 mg/kg). After a single intraperitoneal injection of 200 *μ*L (15 mg/mL) P2, we monitored the drug concentrations in plasma at 13, 20, 40, 68, 100, 127, and 168 min after administration. In Figure 5, the profile of P2 concentration* versus* time, averaged from five individual mice, was shown. This indicated that intraperitoneal administration produced a quick increase of the P2 concentration in plasma and the average profile indicated that, after 13 minutes, the concentration progressively decreased. This result was in accordance with the survival experiments. At 100 minutes, there was still 1.54 *μ*M or 3.63 *μ*g/mL P2 present in mice plasma, which can effectively inhibit the secreted MMP activity. These pharmacokinetic data are in line with the knowledge of a time window of only 10 minutes after LPS administration and thus fully explain the survival curves obtained with P2. These results also confirmed that peptide P2 entered quickly into the circulation and protected mice from endotoxin shock.

## 4. Discussion

In line with previous knowledge [1–3], intravenous LPS injection will immediately act on neutrophils and stimulate these abundant leukocytes to secrete various effector molecules including MMP-8, MMP-9, and reactive oxygen intermediates, which can activate pro-MMPs via a “cysteine switch” mechanism [19]. Early inhibition of MMP activities with peptides can successfully protect mice from endotoxin shock [8, 9], but little is known about the pharmacological behavior of such peptide inhibitors* in vivo*, after different routes of injection. Similar molar amounts of P2 (90 mg/kg) as those of Regasepin 2 showed a significant protective effect. Furthermore, the effect of decreasing doses of P2 was evaluated and it was found that a dosage as low as 10 mg/kg P2 can still show a significant protective effect. An indirect ELISA method with an anti-P2 polyclonal antibody was developed to detect the plasma concentrations of P2. It was found that the plasma concentrations of P2 quickly decreased and for the dosage of 10 mg/kg P2, effective plasma concentration lasted for only 6 minutes. Even for the dosage of 90 mg/kg P2, effective plasma concentration of P2 lasted less than 30 minutes. These results illustrate the need for immediate medical action, within 10 minutes, in all cases of endotoxinemia and explain partially the failure of therapies and high patient mortality rates of about 35%, even with the best medical cares in the most specialized hospitals. Indirect experimental evidence for this paradigm stems from baboon studies in which serum levels of MMP-9 quickly increased and peaked at 2-3 hours [5]. Pugin et al. confirmed that LPS can induce a rapid (within 20 minutes) release of MMP-9 zymogen in whole human blood [20], and Dubois et al. further confirmed that neutrophils contributed to the increase of pro-MMP-9 [6]. Worth of notice is the effective dose of LPS. In the baboon model [5], whole bacteria were injected and it may take some time to accumulate enough LPS or peptidoglycan to stimulate neutrophils. In the latter case, during the gelatin zymography analysis of conditioned plasma after LPS treatment, the LPS dosage was 100 pg/mL and 1 ng/mL. The time for pro-MMP-9 levels to peak after LPS treatment was 1 hour for 100 pg/mL LPS treatment and only 20 minutes for 1 ng/mL LPS treatment. In our endotoxin shock model, 200 *μ*g LPS was intravenously injected into mice. Inhibition of MMP activities within 11 minutes (a six-minute degrading time and a five-minute interval between LPS and peptide administration) is enough to protect mice from lethal endotoxin shock in this experimental setting. Our data, unfortunately, also demonstrate the limited time interval to act in clinical settings.

Second, the MMPs degranulated by neutrophils, for example MMP-8 and MMP-9, are released proenzymes. During endotoxin shock syndrome development, pro-MMP-8 and pro-MMP-9 may be activated by reactive oxygen intermediates [7]. These reactive oxygen intermediates (ROS) have a very short lifetime in plasma and they can be easily neutralized by reducing agents. P2 with a free cysteine in its sequence possesses reducing properties. Even if P2 would be cleaved by abundant proteases, the cysteine residue would still exhibit its reducing activities. Third, the Inhibitor 2 part of P2 also inhibits MMP-8 and MMP-9 activities [11], even if the ES-2 part of P2 would be cleaved off (Figure 1(d)).

The half-life of peptide HM-3 that also contains the ES-2 sequence (see Table 1) was determined to remain only 27 minutes in the rat circulation [12]. The pharmacokinetics of P2 by intraperitoneal injection was also investigated. The peak level of P2 by intraperitoneal injection was reached within 13 minutes (Figure 5). After intraperitoneal injection, P2 entered quickly into the circulation and thereafter its plasma concentration gradually decreased. At 100 minutes after ip injection, the plasma concentration was decreased to 1.54 *μ*M, which means that 90 minutes might be the largest therapeutic interval for P2 at this dosage. Future developments may improve these pharmacological parameters. For instance, the pharmacokinetic character of peptides can be improved by polyethylene glycol (PEG) modification. HM-3 (for sequence, see Table 1) is an antitumor peptide with a half-life of 27 minutes in the rat circulation [12]. After N-terminal modification of the peptide with a PEG molecule of 20 kDa, PEG-HM-3 showed a clearance half-life of 20 hours [21, 22]. Although, after modification of P2 with PEG in a similar way, the PEG-P2 may have a longer plasma half-life and easily pass through blood vessels and get into tissues, the aspect of a limited therapeutic time window will remain an issue. However, many other inflammatory diseases exist with larger time windows [23] and these might benefit from the new insights presented here.

In conclusion, endotoxin shock is a life-threatening immunopathological disorder and early inhibition of MMPs, which are degranulated by neutrophils after LPS stimulation, protects mice from death. Furthermore, the present study defined the strictly limited therapeutic time window in the event of lethal endotoxin shock induction. This information not only is interesting from an immunopathological point of view but also has clinical consequences.

## Figures and Tables

**Table 1:** Peptide sequences and their IC50 values in *μ*M against target enzymes.

Peptide name	Peptide sequence	MMP-8	MMP-9	TACE	Reference
Regasepin 2	Pro-Pyr-Cys-Bip-Arg-Gly-Glu	10	0.8	1.5	[9]

Regasepin 1	Pro-Arg-Cys-Bip-Cys-Gly-Glu	3	1.5	5	[8]

Inhibitor 2	Pro-(D-Pyr)-(D-Cys)-Bip-Arg-Gly-Glu	0.8	2.75	5.5	[11]

ES-2	**Ile-Val-Arg-Arg-Ala-Asp-Arg-Ala-Ala-Val-Pro**	>500	>500	>500	[10]

P2	Pro-(D-Pyr)-(D-Cys)-Bip-Arg-Gly-Glu-Gly-Gly-Gly-Gly-**Ile-Val-Arg-Arg-Ala-Asp-Arg-Ala-Ala-Val-Pro**	0.35	1.36	1.95	[10]

HM-3	**Ile-Val-Arg-Arg-Ala-Asp-Arg-Ala-Ala-Val-Pro**-Gly-Gly-Gly-Gly-Arg-Gly-Asp	ND	ND	ND	[12]

**Table 2:** Peptide treatment strategy for the animal experiments in Figure 1.

Group	Peptide administration strategy
G1 (*n* = 12 or 15)	LPS (100 *μ*L^*^, iv)
G2 (*n* = 12)	LPS (100 *μ*L, iv), 5 minutes later, Regasepin 2 (100 *μ*L^**^, iv)
G3 (*n* = 12)	LPS (100 *μ*L, iv), 5 minutes later, P2 (100 *μ*L^***^, iv)

^*^LPS is dissolved in sterile 0.9% saline at a concentration of 2 *μ*g/*µ*L. ^**^Regasepin 2 was dissolved in sterile 0.9% saline at a concentration of 7 mg/mL. ^***^Peptide is dissolved in sterile 0.9% saline at a concentration of 17.7 mg/mL for the experiment in Figure 1(a), 5.9 mg/mL for Figure 1(b), and 1.97 mg/mL for Figure 1(c).

**Table 3:** Peptide treatment strategy for the animal experiment in Figure 4(a).

Group	Peptide administration strategy
G1 (*n* = 16)	LPS (100 *μ*L^*^, iv),
G2 (*n* = 12)	LPS (100 *μ*L, iv), 5 minutes later, P2 (100 *μ*L^***^, iv)
G3 (*n* = 12)	LPS (100 *μ*L, iv), 5 minutes later, P2 (100 *μ*L, iv), 2 hours later, P2 (100 *μ*L, iv)

^*^LPS is dissolved in sterile 0.9% saline at a concentration of 2 *μ*g/*µ*L. ^***^Peptide P2 is dissolved in sterile 0.9% saline at a concentration of 2.0 mg/mL.

**Table 4:** Peptide treatment strategy for the animal experiment in Figures 4(b), 4(c), or 4(d).

Group	Peptide administration strategy
G1 (*n* = 12 or 15)	LPS (100 *μ*L^*^, iv)
G2 (*n* = 12)	LPS (100 *μ*L, iv), 5 minutes later, Regasepin 2 (100 *μ*L^**^, iv)
G3 (*n* = 12)	P2 (200 *μ*L^#^, ip), wait for some time^##^, LPS (100 *μ*L, iv)

^*^LPS is dissolved in sterile 0.9% saline at a concentration of 2 *μ*g/*µ*L. ^**^Regasepin 2 was dissolved in sterile 0.9% saline at a concentration of 7 mg/mL. ^#^The concentration of P2 was 15 mg/mL; ^##^the time interval was 30, 60, or 90 minutes for the experiments shown in Figures 4(b), 4(c), or 4(d).
